# Strengthening Process by Electron Beam to Carbon Fiber for Impact Strength Enhancement of Interlayered Thermoplastic-Polypropylene Carbon Fiber Composite

**DOI:** 10.3390/ma15217620

**Published:** 2022-10-30

**Authors:** Hideki Kimura, Keisuke Takeda, Helmut Takahiro Uchida, Michael C. Faudree, Kohei Sagawa, Satoru Kaneko, Michelle Salvia, Yoshitake Nishi

**Affiliations:** 1 Graduate School of Engineering, Tokai University, Hiratsuka 259-1292, Japan; 2 Graduate School of Science & Technology, Tokai University, Hiratsuka 259-1292, Japan; 3 Faculty of Liberal Arts and Science, Tokyo City University, Yokohama 224-8551, Japan; 4 Kanagawa Institute of Industrial Science and Technology (KISTEC), Ebina 243-0435, Japan; 5 Laboratory of Tribology and Dynamics of Systems (LTDS) Ecole Centrale de Lyon, CEDEX, 69134 Ecully, France

**Keywords:** composite, thermoplastic, polypropylene, carbon fiber, electron beam, impact value

## Abstract

Strong adhesion between recyclable thermoplastic (TP) polymer and carbon fiber (CF) has always been highly sought after. Therefore, for an interlayered CF reinforced TP polypropylene (CFRTPP) composite composed of 3 sized CF plies, alternating between 4 PP sheets, designated [PP]_4_[CF]_3_, a process of activating CF plies directly on both sides with homogeneous low energy electron beam irradiation (EBI) under N_2_ gas, prior to lamination assembly and hot press of 4.0 MPa at 493 K for 3 min was carried out. Experimental results showed EBI dose of 43.2, 129, or 216 kGy significantly raised Charpy impact values, *a*_uc_ at all fracture probabilities, *P*_f_. The 129 kGy dose appeared to be at or near optimum increasing *a*_uc_ 103%, 83%, and 65% at low-, median-, and high-*P*_f_ = 0.07, 0.50, and 0.93; while raising statistically lowest impact value, *a*_s_ at *P*_f_ = 0 calculated by 3-dimensional Weibull equation about 110%, indicating increased safety and reliability. It is assumed dangling bonds generated by the EBI rapidly form covalent bonds CF:C:O:C:PP and CF:C:C:PP at the interface, along with cross-linking in the PP near the CF. This is by charge transfer from CF to PP.

## 1. Introduction

There has been an urgent need to transition to a more sustainable society through increasing the use of recyclable materials. Carbon fiber reinforced polymers (CFRPs) have long been utilized for space, air, land, and sea vehicles, wind turbines, along with building construction and sports equipment. This is due to them having high strength to weight ratio, strong corrosion resistance and easy formability into different shapes. Typical CFRPs are made with epoxy polymer which has strong adhesion to CF. However, epoxy is a thermoset (TS) that solidifies by cross-linking, making a tight network that cannot be melted, rendering epoxy CFRPs non-recyclable. In addition, the solidification of epoxies takes a relatively long time, consuming significant energy. Thermoplastics (TPs) [1], on the other hand, are recyclable since their solidification mechanism is by molecular interactions rather than crosslinking, and solidification time is typically much less than TSs. TPs can be re-melted and re-solidified to decrease waste with high concern for our environment.

**Figure 1:**
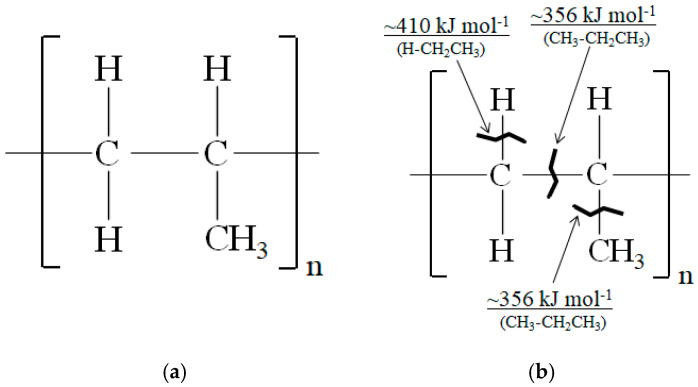
(**a**) Constitutional formula of polypropylene (PP); (**b**) PP showing approximate bonding dissociation energies and dangling bonds [36,37] by charge transfer from EBI−activated CF.

Polypropylene (PP), depicted in Figure 1a is a widely used TP polymer that is inexpensive. Since the short solidification cycle of TPs advances cost reduction, glass fiber reinforced thermoplastic polymers (GFRTPs) [2,3] have been used in automobile manufacture. On the other hand, TPs and CF are difficult to adhere due to their being nonpolar, having low wettability, and hydrophobicity with their chemically inert surfaces [4].

Therefore, a wide body of research has been focusing on CF surface treatments to strengthen adhesion at the CF/Polymer interface [4,5,6,7,8,9,10,11,12,13,14,15,16,17,18,19,20,21,22,23,24,25,26,27,28,29,30,31,32,33,34,35]. One method used is to create higher interfacial friction at the CF surface by acidic modification [5], however, drawbacks are lowered strength [6,7] by degradation of the surface, along with weight reduction of the fiber itself [8]. Superheated steam for 1 h at 650 °C (K) has been used on recycled CF to increase adhesion to PP resin by addition of functional groups containing oxygen [9].

CF surface modifications by applying plasma have been extensively researched [10,11,12] and have been found to enhance CFRP interlaminar shear strength. Interfacial shear stress in epoxy CFRP is reported to be increased about 7 times by plasma oxidation treatment to the CF surface [13].

Since CF and TP surfaces are nonpolar, several studies have focused on creating polar groups on the CF surface [4,14,15,16]. These include oxygen attachment [4], electro-polymer coating generating polar functional groups by chemical grafting [14]; and attachment of rare earth particles [15,16].

On the other hand, radiation processing technology has been commonly utilized for cross-linking, grafting, or curing materials for aerospace [17], automobile, construction, and heath care [18,19]. High energy irradiation treatments such as ions and γ-rays to CF have been used to increase CF/Polymer adhesion [20,21], stimulating active sites in the CF crystal lattice and enhancing surface roughness. Examples have included Ar^+^ [22] and Co-60 γ-ray [23] irradiation. γ-ray treatments have energy of ~1.25 × 10^6^ eV, and have deep penetration depth, suitable for high density materials. However, absorbed dose rate is low, so longer irradiation times are required [24]. Moreover, use of γ-ray has safety considerations in the treatment facility.

Therefore, lower energy electron beam irradiation (EBI) (10^2^ to 10^3^ eV) has been preferred, due to having the advantage of higher dose rate leading to shorter irradiation times. Other advantages are, uses no chemicals, can treat large parts, and low cost. EBI to the inert CF surface improves wettability [25], and generates polar groups to increase adhesion [24,26].

EBI has increased mechanical properties of CFRPs of both TS epoxies [27,28,29] and TP [30]. Applying EBI to PEEK CFRTP samples increased Charpy impact values 56% at low fracture probability, *P*_f_ over untreated increasing reliability and safety [30].

EBI has been applied to both outside surfaces of finished CFRP samples [27,28,30], or directly to CFs in samples prior to assembly and heating [31] enhancing strength. EBI has been used with plasma polymerization to increase adhesion strength of CFRP [32], and for grafting on the CF surface [33]. Radiation cross-linker has been used in conjunction with EBI. In a PP CFRTP, triallyl isocyanurate (TAIC) with 100, 200, or 400 kGy EBI dose in air atmosphere substantially increased tensile strength over: EBI irradiated without TAIC, and untreated [34].

Our previous research has shown in a thermoplastic [PP]_4_[CF]_3_ interlayered composite, direct activation of 0.22 MGy EBI to CF plies prior to lamination assembly and hot press slightly increases maximum bending strength, *σ*_b_ about 6% at median-*P*_f_ of 0.50 [35]. However, the effect of EBI on the essential mechanical property of impact strength has not been investigated. Hence, the goal of this study is to demonstrate Charpy impact values of [PP]_4_[CF]_3_ can be improved by EBI directly to CFs.

Note for sustainability evaluation of a product, Life Cycle Assessment (LCA) is carried out. This means calculating the carbon footprint from extraction of resources from the Earth to disposal or recycle. High energy treatments to CF are only one step of the fabrication process, but must be taken into account since they can alter the carbon footprint. However, LCA analysis is beyond the scope of this study.

## 2. Materials and Methods

### 2.1. Samples

Samples were an interlayered CF reinforced TP polypropylene (CFRTPP) composite composed of 3 sized CF cross-weave plies (TR3110M; Mitsubishi Rayon Ltd. Tokyo; plain weave; areal weight listed by manufacturer is 200 gm^−2^), alternating between 4 PP sheets (BC06C Novatec, Nissho Ltd., Tokyo, Japan), designated [PP]_4_[CF]_3_. Presence and composition of CF sizing was confirmed by proton-NMR (AVANCE500, Neutron Magnetic Resonance, Shimazu, Kyoto, Japan) [35].

**Figure 2:**
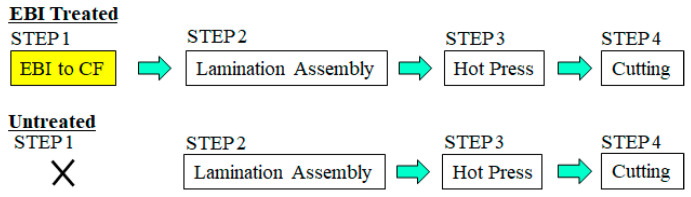
Specimen fabrication process.

Samples were homogeneous electron beam irradiation (EBI) treated, and untreated. Fabrication was 4 basic steps as shown in Figure 2.

**STEP 1:** EBI treatment of single CF plies on both sides prior to lamination assembly (see Section 2.2) Untreated samples skip this step (Figure 2).

**STEP 2**: Lamination assembly of CF with PP into [PP-CF-PP-CF-PP-CF-PP] layup.

**STEP 3**: Solidification of the CFRTPP [PP]_4_[CF]_3_ by one directional hot-press (IMC-185A, Imoto Machinery Co., Ltd., Tokyo, Japan) under 4.0 MPa at 493 K for 3 min.

**STEP 4**: Samples were cut into dimensions length (*l*), width (*w*) and thickness (*t*) of: 80 mm × 10 mm × 2 mm. 

Finished samples were approximately 55% CF by volume. It is assumed the thickness of each ply in the [PP]_4_[CF]_3_ composite finished samples is 2.0 mm/7 = 286 μm. Samples will be referred to herein as “CFRTPP” or “[PP]_4_[CF]_3_”.

### 2.2. Condition of EBI

An electron-curtain processor (Type CB175/15/180L, Energy Science Inc., Woburn, MA, USA, Iwasaki Electric Group Co., Ltd., Tokyo, Japan) [4] was used to homogeneously irradiate both sides of the CF plies. To obtain total EBI irradiation dose, CF plies were swept back and forth under the electron beam. One sweep going one way was 43.2 kGy taking 23 s, with 30 s between each interval to avoid overheating. The CFs were irradiated in protective N_2(g)_ atmosphere with residual O_2(g)_ concentration less than 300 ppm. Table 1 summarizes parameters and settings used.

Irradiation dose was controlled by integrated irradiation time for each sample, and corrected by using an FWT nylon dosimeter of RCD radiometer film (FWT-60-00: Far West Technology, Inc., 330-D South Kellogg Goleta, CA 93117, USA) with an irradiation reader (FWT-92D: Far West Technology, Inc., 330-D South Kellogg Goleta, CA 93117, USA). For more details of EBI used, see Kitagawa et al. (2019) [4].

Based on mean density (*ρ*: kg/m^3^) and irradiation potential at specimen surface (*V*: keV), penetration depth (*D*_th_: /m) of EBI is expressed by the Christenhusz and Reimer equation [38]:

*D*_th_ = 66.7*V*
^5/3^/*ρ* (1)

Given CF density, *r*_CF_ of 1760 kgm^−3^, *D*_th_ is estimated to be 123 μm [30]. The 66.7 is a constant with units (μm) (kgm^−3^) (keV^−5/3^). 

Dangling bonds naturally exist in CF as evidenced by electro-spin resonance (ESR) peak generation whose inflection point is at *B* = ~323 mT [30]. However, in the highly conductive CFs, the ESR peak is reduced by EBI indicating CF dangling bond reduction, resulting in strengthening of the CF [30]. The activated CF plies should transfer charge to the PP plies to increase adhesion and strength of the CFRTPP composite. Figure 1b shows dangling bond locations in PP with approximate bonding dissociation energies [36,37].

### 2.3. Charpy Impact Test

EBI treated and untreated [PP]_4_[CF]_3_ samples were tested using a pendulum type impactor (No. 51735, Shimadzu Corporation, Tokyo, Japan) (JIS K 7077) [2] to obtain Charpy impact values, *a*_uc_. Impact fracture energy, *E* (kJ) is expressed by the following equation [2]:

*E* = *WR*[(cos*β*-cos*α*) − (cos*α*′-cos*α*)(*α* + *β*)/( *α* − *α*′)] (2)

Here, *W*, *R*, *β*, *α* and *α*′ are hammer mass (0.86 kg), length (0.21 m) of hammer weight point from rolling center, maximum angle after impact (Radians), start angle before impact (*α* = 2.3 Radians or 132°), and maximum angle of the blank test (Radians), respectively. The *a*_uc_ (kJm^−2^) is expressed by the following equation:

*a*_uc_ = *E*/(*b* × *t*) (3)

Here, *E*, *b* (=10 ± 0.2 mm) and *t* (=2.00 ± 0.15 mm) are impact fracture energy (J), sample width (mm), and sample thickness (mm), respectively. The distance between supporting points was 40 mm.

### 2.4. Cumulative Probability

Cumulative probability (*P*_f_) evaluation is used to rank sample strength and is widely applied to quantitatively analyze data from experiments and in industry. It is often employed in statistical quality control (QC) to assess safety and reliability of manufactured parts. *P*_f_ calculation utilizes a general form of the median-rank method [39]:

*P*_f_ = (*i −* 0.3)/(*N*_s_ + 0.4) (4)

The *N*_s_ and *i* are total number of samples and rank order integer of bending strength of each sample, where *i* is from weakest to strongest. In this study, *N*_s_ = 9, thus, for *i* values 1, 5, and 9, corresponding *P*_f_ values are 0.07, 0.50 and 0.93.

## 3. Results: Effects of EBI to CF on Impact Value of CFRTPP

**Figure 3:**
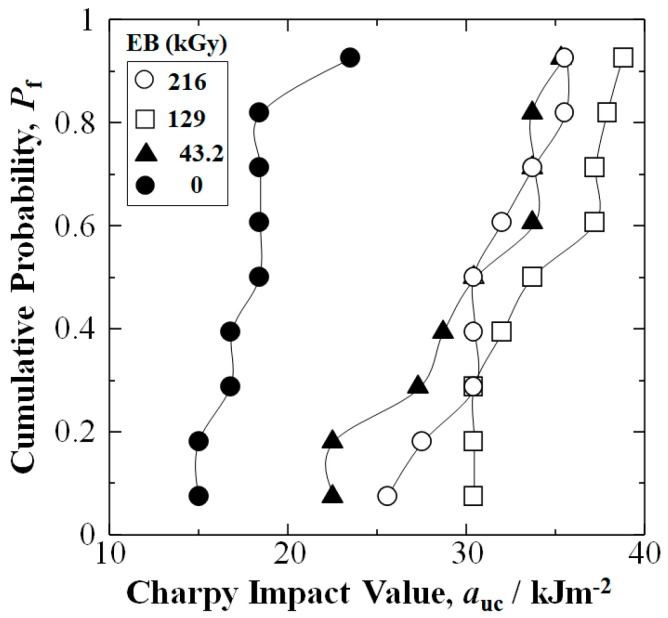
Cumulative probability, *P*_f_ vs. Charpy impact value, *a*_uc_ (kJm^−2^) for untreated, 43.2 kGy, 129 kGy, and 216 kGy data sets.

**Figure 4:**
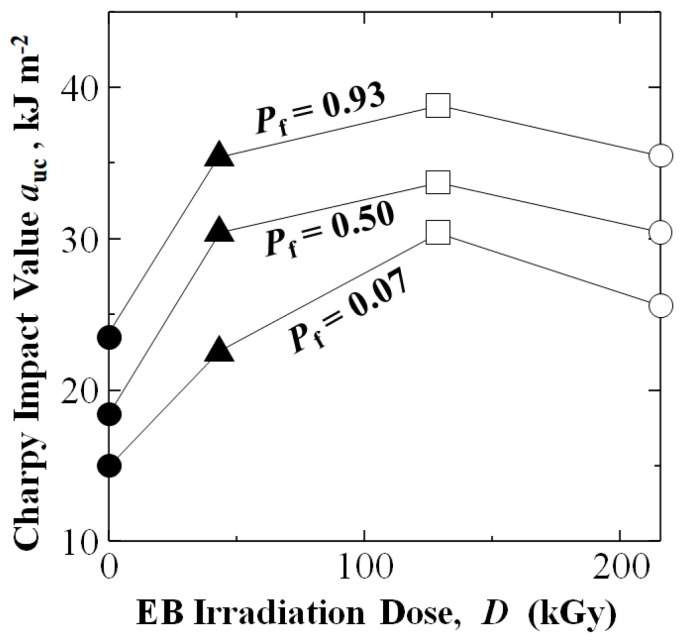
Charpy impact values (*a*_uc_) at low, median and high cumulative probabilities (*P*_f_) of 0.07, 0.50 and 0.93 from the data in Figure 3.

Experimental results in Figure 3 show by applying EBI of 43.2, 129 or 216 kGy to CF, *a*_uc_ of [PP]_4_[EB]_3_ samples were considerably raised at all *P*_f_ over untreated. Figure 4 shows the 129 kGy was at or near the optimum dose, raising Charpy impact values to 30.4, 33.7, and 38.8 kJm^−2^, which were about 103%, 83%, and 65% higher than that of untreated at 15.0, 18.4, and 23.5 kJm^−2^ at low, median, and high *P*_f_ of 0.07, 0.50 and 0.93, respectively.

Moreover, as shown in Figure 4, the 43.2 kGy dose increased *a*_uc_ to 22.5, 30.4, and 35.3 kJm^−2^, increases of 50%, 65%, and 50%: whereas the higher 216 kGy dose increased *a*_uc_ to 25.6, 30.4, and 35.5 kJm^−2^ showing increases of 71%, 65%, and 51%, respectively. Note Figure 3 and Figure 4 show the weakest samples at low *P*_f_ of 0.07 were increased significantly in all EBI data sets: 43.2, 129, and 216 kGy.

## 4. Discussion

### 4.1. Statistically Lowest Impact Value a_uc_ (a_s_) at P_f_ = 0

To obtain the statistically lowest impact value of a data set, *a*_uc_ (*a*_s_) at *P*_f_ = 0, the 3-dimensional Weibull calculation, often used in quality control (QC) is employed [40].

When the equation is assumed to be applicable to the experimental *a*_uc_ values, the *P*_f_ depends on risk of fracture [40]. The *a*_s_, the coefficient, *m*, and the constant, *a*_III_, are key parameters for predicting the required value for new structural materials [40]:

*P*_f_ = 1 − exp[−([*a*_uc_ − *a*_s_]/*a*_III_)^m^] (5)

Changing into linear form [40]:

ln(−ln(1 − *P*_f_)) = *m*ln(*a*_uc_ − *a*_s_) − *m*ln*a*_III_ (6)

**Figure 5:**
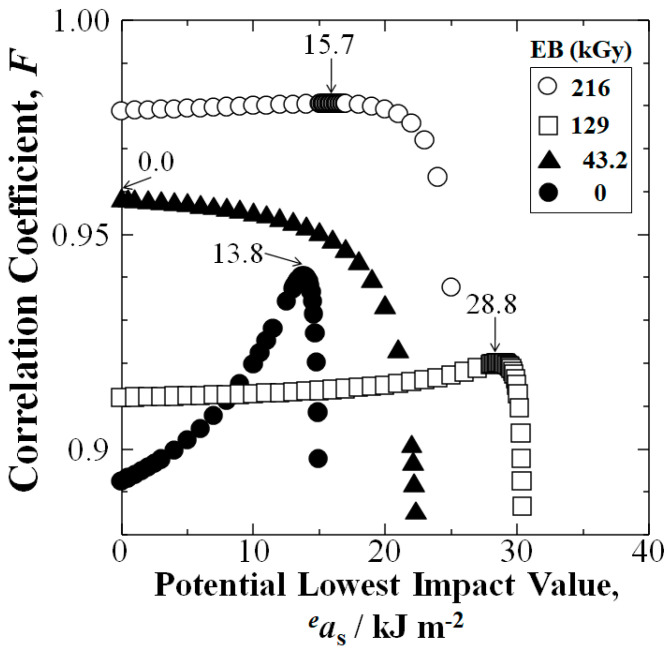
Iteration of potential lowest *a*_s_ value (*^e^a*_s_) to obtain lowest impact value, *a*_s_ at *P*_f_ = 0 (arrows) at maximum correlation coefficient (*F*) for each data set.

Figure 5 shows iteration of Equation (6) for highest correlation value, *F* to obtain the *a*_s_ at *P*_f_ = 0 for the CFRTPP samples. The 129 kGy EBI data set (squares) exhibited the highest *a*_s_ at 28.8 kJm^−2^ over that of untreated at 13.8 kJm^−2^, an increase of 109% indicating increased safety and reliability. Moreover, Figure 5 shows the 216 kGy samples had *a*_s_ of 15.7 kJm^−2^, an increase of 13% over untreated. Although the 43.2 kGy CFRTPP samples had *a*_s_ equal to 0 kJm^−2^, Figure 3 shows the 43.2 kGy EBI dose substantially increased *a*_uc_ at all *P*_f_ over those of untreated.

**Figure 6:**
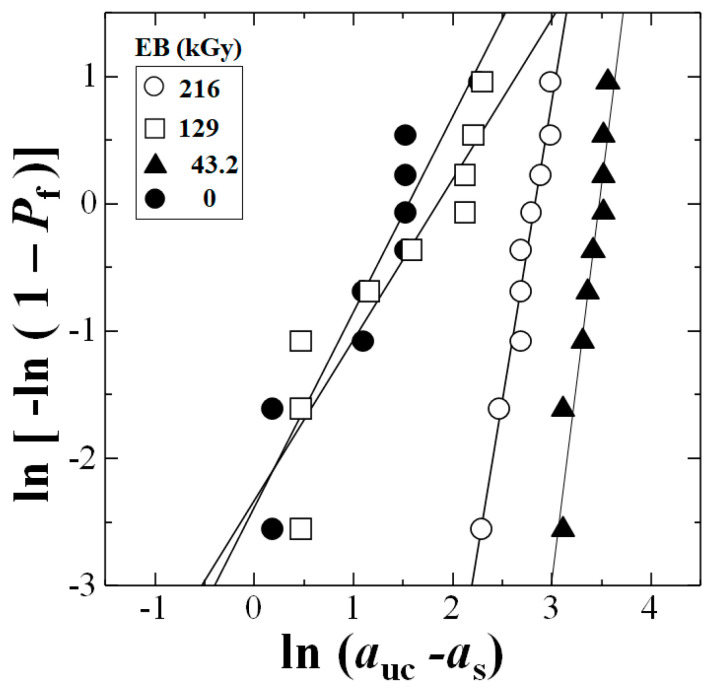
Linear relationships between ln(*a*_uc_ − *a*_s_) and ln[−ln(1 − *P*_f_)] from Weibull 3−D calculation for each data set.

Figure 6 shows the linear plots between ln(*a*_uc_ − *a*_s_) and ln[−ln(1 − *P*_f_)] from Equation (6).

To discuss the validity of the Weibull approach employed here, it is typically used for QC in industry to evaluate new structural materials for safety and reliability [40]. Small changes in experimental scattering can affect the *F* values. However, rather than the absolute *F* values, important is comparing those on the *x*-axis, the statistically lowest value, *a*_s_ at *P*_f_ = 0. For example, the 129 kGy data set yielded the highest *a*_s_ at *P*_f_ = 0 of 28.8 kJm^−2^ indicating the highest safety and reliability.

**Figure 7:**
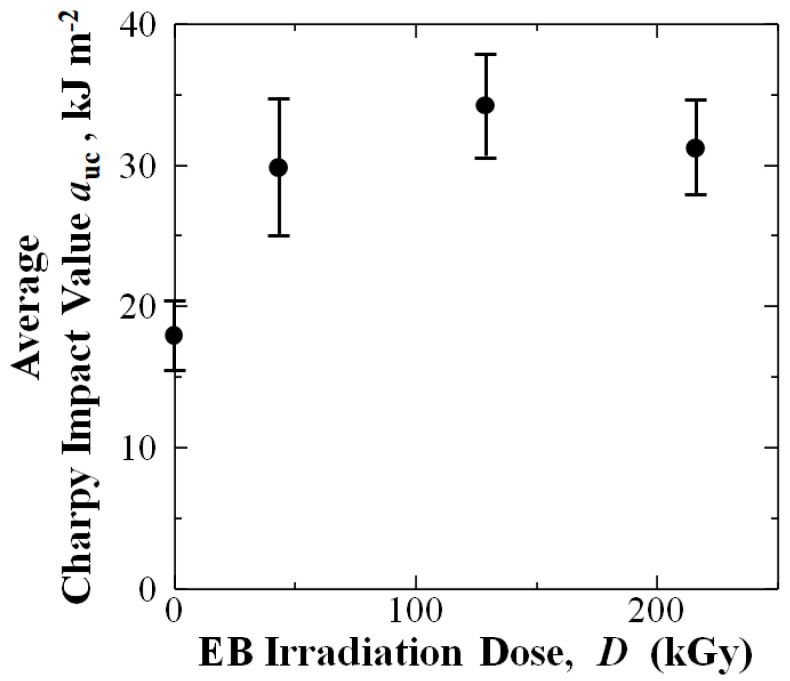
Average *a*_uc_ values with standard deviation bars as a function of EBI dose.

When average *a*_uc_ is plotted against EBI dose, Figure 7 shows standard deviation error bars for the EBI data sets, 43.2, 129, and 216 kGy, are clearly above those of untreated. This indicates EBI doses applied can statistically improve *a*_uc_ of the [PP]_4_[CF]_3_ samples.

Comparison of error bars for 43.2, 129, and 216 kGy data sets in Figure 7 show low variance with 129 kGy condition exhibiting the highest values.

Table 2 shows average *a*_uc_ generally follows the same trend as *a*_uc_ at median-*P*_f_ = 0.50.

### 4.2. Predicted Strengthening Mechanism

**Figure 8:**
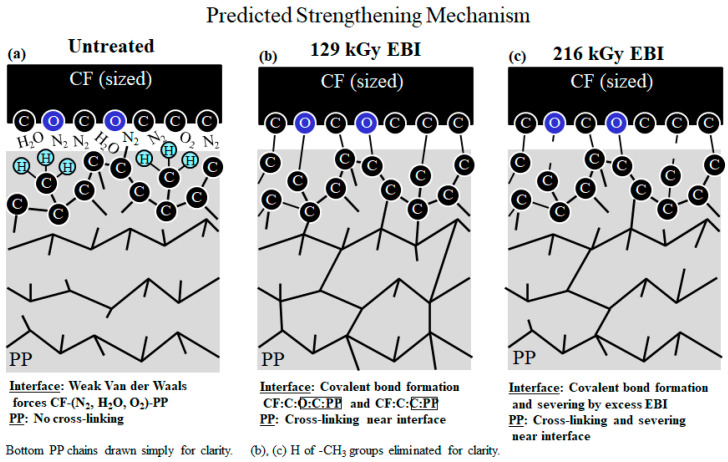
Schematic of CF/PP interface for: (**a**) untreated, (**b**) 129 kGy, and (**c**) 216 kGy EBI samples, respectively. Note: H of -CH_3_ groups eliminated for clarity in (**b**,**c**). O with C atoms included at CF surface to indicate epoxy sizing.

Figure 8 explains the predicted strengthening mechanism at the CF/PP interface by EBI.

**Untreated: **Figure 8a illustrates lower impact value range of 15 < *a*_uc_ < 24 kJm^−2^ (Figure 3) of untreated is predicted to be achieved by weak intermolecular bonding of Van der Waals forces by trace gasses in the EBI chamber of CF-(N_2_, O_2_, H_2_O,)-PP [4,31], along with intermolecular entanglement and copolymerization between CF sizing and PP resin [35] (not shown).

**129 kGy EBI: **However, Figure 8b illustrates the enhancement in impact value range to 31 < *a*_uc_ < 38 kJm^−2^ by 129 kGy EBI is assumed to be by formation of strong covalent bonds CF:C:O:C:PP and CF:C:C:PP at the interface, along with cross-linking into the PP near the CFs by charge transfer into the PP. It is assumed dangling bonds generated by the EBI rapidly form covalent bonds at the interface.

Supporting this, the literature states EBI directly to CF generates excess electrons in the hexagonal structure while increasing strength of CF itself [30]. Part of the excess electron charge is assumed to transfer into the PP. It follows, ESR results have shown PP generates dangling bonds when treated by EBI as increase in peak intensity with inflection point at 322.5 mT [2].

To explain formation of O and C covalent bonds at the interface, previous research confirmed X-ray photoelectron spectroscopy (XPS) detected C=H; C-C; C-O; C-O-C=O; C=O; and COOH bonds at CF surface with removed sizing [26]; while typical epoxy CF sizing has -OH groups. Moreover, Jung et al. (2020) reported for high-density polyethylene (HDPE) CFRTP samples, spectrographic analysis showed EBI-treatment generated O groups on the CF and HDPE surfaces, along with cross-linking in the HDPE near the CF acting to increase tensile strength. They speculated strengthening at the interface was by strong intermolecular forces between polar O groups along with cross-linking [41]. Single CF tensile tests showed EBI to CF surface enhanced interfacial shear strength (IFSS) reducing fiber pull-out in (HDPE) CFRTP [41].

To date, there is no direct evidence covalent bonds are formed at the CF/PP interface under the experimental conditions herein. However, formation of covalent bonds has been used to explain strengthening of C/C composites [42].

As mentioned earlier, EBI penetration depth of CF is *D*_th_ of 123 μm on both side surfaces. Hence, the 286 μm CF ply thicknesses would represent [skin-core-skin] sandwich structures of: [123/40/123] μm = [*D*_th_/40/*D*_th_]. This allows each CF ply to be well-activated for adhesion to neighboring PP plies throughout the [PP]_4_[CF]_3_ specimen thickness.

**261 kGy EBI: T**he 129 kGy EBI dose appears to be at or near optimum. This is because higher dose of 216 kGy decreases *a*_uc_ range to 25 < *a*_uc_ < 35 kJm^−2^. Dangling bonds in PP are reported to increase with increasing EBI dose as increase in ESR peak intensity [2]. Thus, the mechanism is predicted to be covalent bond formation with bond severing by excess EBI at the interface, and cross-linking with severing in the PP near the CF as illustrated in Figure 8c.

As for maximizing CF/PP adhesion area, optimum EBI dose apparently increases sparse and nonhomogeneous crystallization sites around CF circumference [35]. The highly conductive CF allows even distribution of electric charge over the entire CF surface to expand the number of adhesion sites.

As mentioned earlier, dangling bond density in CF is reduced by EBI. For instance, it is reported 0.43 MGy EBI lowers dangling bond density from 1.15 × 10^18^ spins-m^−3^ to 0.78 × 10^18^ spins-m^−3^ [30]. However, quantification of dangling bonds or electric charge transferring from the CF to PP is beyond the scope of this study.

## 5. Conclusions

In a carbon fiber reinforced thermoplastic polypropylene (CFRTPP) interlayered composite composed of 3 CF mats between 4 PP (polypropylene) sheets, [PP]_4_[CF]_3_, impact values were increased by applying homogeneous low energy electron beam irradiation (EBI) directly to CF plies prior to lamination assembly and hot-press.

1. Experimental results showed EBI dose of 43.2, 129, or 216 kGy improved Charpy impact values, *a*_uc_ at all cumulative probabilities, *P*_f_ over untreated.
2. The 129 kGy-EBI doses yielded the highest *a*_uc_; hence, is considered at or near the optimum. The 129 kGy-EBI raised *a*_uc_ to 30.4, 33.7, and 38.8 kJm^−2^, which were about 103%, 83%, and 65% higher than that of untreated at 15, 18, and 23.5 kJm^−2^ at low, median, and high *P*_f_ of 0.07, 0.50 and 0.93, respectively.
3. The 129 kGy EBI dose increased the statistically lowest impact value *a*_s_ at *P*_f_ = 0 calculated by 3-dimensional Weibull method about 110% over untreated indicating increased safety and reliability.
4. It is assumed improvements in *a*_uc_ result from formation of maximum number of strong covalent bonds CF:C:O:C:PP and CF:C:C:PP at the CF/PP interface, along with cross-linking in the PP near the CF/PP interface.
5. Higher EBI dose of 216 kGy lowering the *a*_uc_ is predicted to occur by covalent bond generation accompanied by radiation damage in the form of bond severing at the CF/PP interface; along with cross-linking with severing in the PP near the interface. Therefore, EBI dose must be adjusted carefully when using for practical parts.

## Figures and Tables

**Table 1:** EBI parameters and settings.

Parameter	Setting	Parameter	Setting
Linear electron gun	Ti filament	EBI dose/Sweep	43.2 kGy
Acceleration potential	170 kV	Time/Sweep	23 s
Current density	0.089 Am^−2^	Conveyor speed	10 m min^−1^
Distance Sample and Ti window	25 mm	Time between sweeps	30 s
Sample atmosphere	N_2_ (<300 ppm O_2_)	*T*_max_ of sample	323 K
N_2_ flow rate and P	1.5 Ls^−1^, 0.1 MPa		

**Table 2:** Average *a*_uc_ values (standard deviations in brackets) with *a*_uc_ at median *P*_f_ = 0.50 as a function of EBI dose. Units of *a*_uc_ in kJm^−2^.

	Untreated	43.2 kGy	129 kGy	216 kGy
avg. *a*_uc_ (std. dev.)	17.9 (2.5)	29.8 (4.9)	34.2 (3.6)	31.2 (3.4)
median *a*_uc_ at *P*_f_ = 0.50	18.4	30.4	33.7	30.4

## Data Availability

The data presented in this study are available on request from the corresponding author. At the time the project was carried out, there was no obligation to make the data publicly available.
